# Antibiotic Susceptibility Patterns and Molecular Epidemiology of Metallo-β-Lactamase Producing Pseudomonas Aeruginosa Strains Isolated From Burn Patients

**DOI:** 10.5812/ircmj.10916

**Published:** 2014-05-05

**Authors:** Aziz Japoni, Mojtaba Anvarinejad, Shohreh Farshad, Giovanni M Giammanco, Noroddin Rafaatpour, Ebrahim Alipour

**Affiliations:** 1 Alborzi Clinical Microbiology Research Center, Nemazee Teaching Hospital, Shiraz University of Medical Sciences, Shiraz, IR Iran; 2 Department of Health Promotion Sciences, University of Palermo, Palermo, Italy

**Keywords:** Beta-Lactamases, Imipenem, Meropenem, Pulsed Field Gel Electrophoresis

## Abstract

**Background:**

Failure in the treatment of burn patients infected with Pseudomonas aeruginosa could happen as a result of the acquisition of antibiotic resistance, including carbapenems.

**Objectives:**

The aim of the present study was to investigate the phenotypic and genotypic characteristics of the Pseudomonas aeruginosa strains, isolated from burn patients.

**Patients and Methods:**

During a 12 month period, in this cross-sectional study, two hundred seventy strains of Pseudomonas aeruginosa were isolated from the burn patients in Ghotbeddin Burn Hospital, Shiraz, Iran. Screening for the carbapenem resistance in the isolates was carried out by the E test method. Sensitivity patterns of metallo-β-lactamase (MβLs) producing strains of pseudomonas to eleven antibiotics were determined by the mentioned method. The epidemiological associations of these strains were determined by Pulsed-field gel electrophoresis (PFGE).

**Results:**

Of the 270 strains, 60 (22.2%) were resistant to imipenem and meropenem, classified as MβLs producing. MβLs producing strains of pseudomonas were completely resistant to five tested antibiotics while their sensitivities to the three most effective antibiotics including ceftazidime, amikacin and ciprofloxacin were 23.4%, 6.7 % and 1.7%, respectively. In PFGE, 37 patterns from the genome of Pseudomonas aeruginosa were observed. Majority of the strains (43; 71.6%) exhibited more than 80% similarity, based on the drawn dendrogram.

**Conclusions:**

According to the results, none of the tested antibiotics is safe to prescribe. As PFGE revealed, a limited number of Pseudomonas aeruginosa types are predominant in the hospitals which infect the burn patients.

## 1. Background

*Pseudomonas aeruginosa* (PA) is one of the leading pathogens responsible for nosocomial infections and occurs so frequently in the environment that it is very likely an individual suffering from severe burns will face therapeutic challenges due to the infection with this microorganism before the burns can heal (1, 2).

Burn hospitals are often exposed to multidrug-resistant *Pseudomonas aeruginosa* (MRPA) that can act as the source of infections. *P. aeruginosa* has been found to contaminate the floors, bed rails, and sinks in the hospitals, and has been cultured from the hands of nurses, as well (3, 4). Phenotypic and genotypic methods are increasingly being used to monitor the source and transmission routes of diseases, as well as the emergence of the strains with increased pathogenicity. Among the various available methods for molecular and epidemiological detection of the microorganisms, Pulsed-field Gel electrophoresis (PFGE) has gained more attention because of its high discriminatory power and reproducibility and therefore, is introduced as the gold standard in molecular and epidemiological investigations (5-7). The growing prevalence of infections by MRPA is associated with a significant morbidity and mortality due to the lack of alternative treatment (8). Some recently reported studies support the use of colistin and tigecycline, as the effective agents against MRPA. However, their administration in the clinics is limited because of their adverse effects and reported resistance (9, 10). Carbapenems are one of the effective antimicrobial agents used to treat the infections caused by PA with extended spectrum β-lactamase activity (11). In recent years, several nosocomial outbreaks of metallo-β-lactamase (MβLs) of PA have been reported across the world which were associated with carbapenem resistance and different types of integron class 1 (12-15). A limited number of studies in Iran have attempted to characterize burn patients with MβLs producing PA on the epidemiological and molecular basis and antibiotic susceptibility.

## 2. Objectives

This epidemiological study was designed to investigate the reliability of antibiogram and pulsed field gel electrophoresis in detecting the genetic patterns, relationship and clonality of the PA strains, isolated from burn patients.

## 3. Patients and Methods

In this cross-sectional study conducted from April 2009 to April 2010, a total of 270 samples of PA were taken from burn patients in Ghotbeddin Burn Hospital, Shiraz, Iran. Data about gender, age, duration of hospitalization, cause, site, degree and types of burns (accidental or suicidal) from the patients infected with MβLs producing pseudomonas were collected through questionnaires filled by the professional nurses. Total body surface area (TBSA) was estimated by the rule of nines (16) and the degree of burns were determined by the depth of injury (17). Informed written consents were obtained from the patients enrolled in the study. The design and protocol of the study were also approved by the ethics committee of Professor Alborzi Clinical Microbiology Research Center.

### 3.1. Bacterial Isolation

Specimens were collected by sterile swab after the removal of dressing and cleansing of the wound surface with 70% alcohol. Sampling was accompanied by mild pressure of swab to cause bleeding in the underlying tissue.

The burn samples were cultured on nutrient agar media (Oxoid) and incubated overnight at 37^◦^C. Any suspicious colony was then subcultured and purified. The isolates were identified as *P. aeruginosa *based on oxidase test, triple sugar iron (TSI) fermentation, color, pyocyanin pigment production and odor. Strains were preserved at -70^◦^C on nutrient broth No2 (Oxoid) containing 30% (v/v) glycerol. As the cases considered in this study were only MβLs producing pseudomonas, the inclusion criteria were the strains resistant to IMI and MEM, known as MβLs.

### 3.2. Phenotypic Detection of MβLs

MβL E test strips (AB Biodisk, Sweden) were used to screen class B β-lactamase. Tests were carried out and interpreted according to the manufacturer's instruction.

### 3.3. Antibiotic Susceptibility Patterns

Minimum inhibitory concentrations (MICs) of the 11 antibiotics, routinely prescribed in burn centers, against the 60 isolates of MβLs producing *P.aeruginosa*, were determined by the E test method (AB Biodisk, Sweden) as recommended by the National Committee for Clinical Laboratory Standard (CLSI) (18). These antibiotics included: imipenem (IMI), meropenem (MEM), cefepime (CPM), ceftazidime (CAZ), piperacillin/tazobactam (PTZ), ciprofloxacin (CIP), tobramycin (TN), amikacin (AK), gentamicin (GM), ampicillin (AP) and aztreonam (ATM). American typing collection (ATCC 27853) of *P. aeruginosa *was used as a control strain in antibacterial susceptibility determination. Following overnight incubation in Muller-Hinton agar, the MICs breakpoints were interpreted according to the manufacturer’s instructions.

### 3.4. Pulsed-Field Gel Electrophoresis

This procedure was designed based on previously reported protocol by Ejnreas et al. (19) with some modifications, as described. PFGE with the restriction enzyme XbaI was performed in duplicate for the total genome of some strains to determine the reproducibility of the equipment. The electrophoretic conditions used, were as follows: initial switch time, 5 seconds; second switch time, 20 seconds; final switch time, 40 seconds; temperature 6^◦^C, run time 33 hours; angle 120^◦ ^and the gradient, 6 v/cm. In each set 1000 bp lambda ladder (Biolabs, New England) was used as molecular marker. PhotoCapt software (VilberLoumart, Marnesla Valle, France) was used to determine the molecular weights of the sample profiles. The bands movement on the gel were compared with the DNA marker and were scored across all the samples. They were recorded as number one for “present” or zero for “absent”. Subsequently, the data were used to calculate pair-wise similarity coefficient following the Jaccard method. To generate a dendrogarm using average linkage procedure, the analysis of the similarity coefficients matrices was performed using unweighted pair-group method analysis (UPGMA). To calculate correlations among the variables, the standardized data matrices were used. The correlations were subjected to Eigenvector analysis to evince the first three uttermost elucidative principal components. To study the patterns of variations observed among the isolates, the three principal components were plotted. The software NTSYSpc version 2.02i (Exeter software, New York) was used to conduct all the numerical analysis. Data analysis was performed using SPSS software, ver. 15 (SPSS, IBM, USA). Chi-square was used to compare the categorical variables. P values less than 0.05 were considered significant. The results were expressed as frequency (percentage).

## 4. Results

### 4.1. Patients’ Data

Of the 270 isolates, 60 (22.2%) were classified as MβLs producing isolates. Male/female ratio was 29/31. Patients aged between 21 to 30 years, were more prevalent (40, 39%), followed by ages 31 to 40 (14, 23%); (Table 1). The burn wounds in the majority of the patients (n = 53, 88.4%), were classified as third degree burns, in terms of severity and extension of burn site. These data are presented in Table 2. The rate of accidental burns in male patients was higher, compared with that of females, while suicidal burns in females especially in women from the rural areas was noticeable. The percentage of burn by flame (93.3%) was significantly higher than electrical and chemical burns (P < 0.001) (Table 2).

**Table 1.** Age Distribution of the Patients Infected With MβLs Producing *P. aeuroginosa*

Age (Range 9-87), y	Values, %
**1-10**	4%
**11-20**	10%
**21-30**	39%
**31-40**	23%
**41-50**	12%
**51-60**	3%
**61-70**	3%
**71-80**	3%
**81-90**	3%

**Table 2.** Demographic data of 60 Burn Patients Infected With MβLs Producing *P. aeuroginosa *^a,b^

Parameters	-
**Age range, y**	9/87
**Male/Female ratio**	29/31
**Accidental**	-
Male	28/48 (58.3)
Female	20/48 (41.7)
**Suicidal**	-
Male	1/12 (8.3)
Female	11/12 (91.7)
**TBSA**	18% - 74%
**Burn site**	-
Whole body except head	17 (28.3)
Whole body	32 (53.3)
Hands	7 (11.7)
Legs	4 (6.7)
**Degree of burn**	-
Type 3	53 (88.4)
Type 2	5 (8.3)
Type 1	2 (3.3)
**Cause of burns**	-
Flame	56 (93.3)
Electrical	3 (5)
Chemical	1 (1.6)
**Living place**	-
Urban	46 (76.6)
Rural	14 (23.4)
Duration of hospitalization, d	1-120

^a^ Abbreviation: TBSA, total body surface area

^b^ Data are presented as No. % or %

### 4.2. Bacterial Isolates and Antibiotic Susceptibility Patterns

Totally, 60 PA which were MβLs producer were collected from the burn patients. These strains were completely resistant to five antibiotics including; imipenem, meropenem, Piperacillin/tazobactam, ampicillin and aztreonam and presented low sensitivity ranging between 1.7% and 23.4% to the rest of the antibiotics. The sensitivity rates were found to be 1.7% for CPM, GM, TN, CIP, 6.7% for AK and 23.4% for CAZ. All the isolates were resistant to three or more antibiotics and were designated as multidrug resistant (MDR).

### 4.3. Pulsed-Filed Gel Electrophoresis

**Figure 1.**
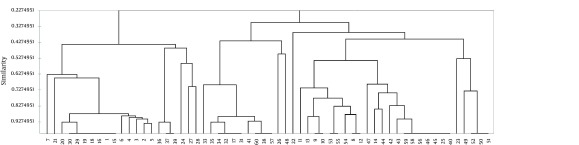
Dendogram and Comparison of 37 Genotype Patterns and 3 Clusters Obtained by PFGE of the Genomic DNA With XbaI Enzyme The similarity among the patterns of the 60 isolates of MBLs was calculated by Dice Coefficient and then regrouped using the UPGMA.

**Figure 2.**
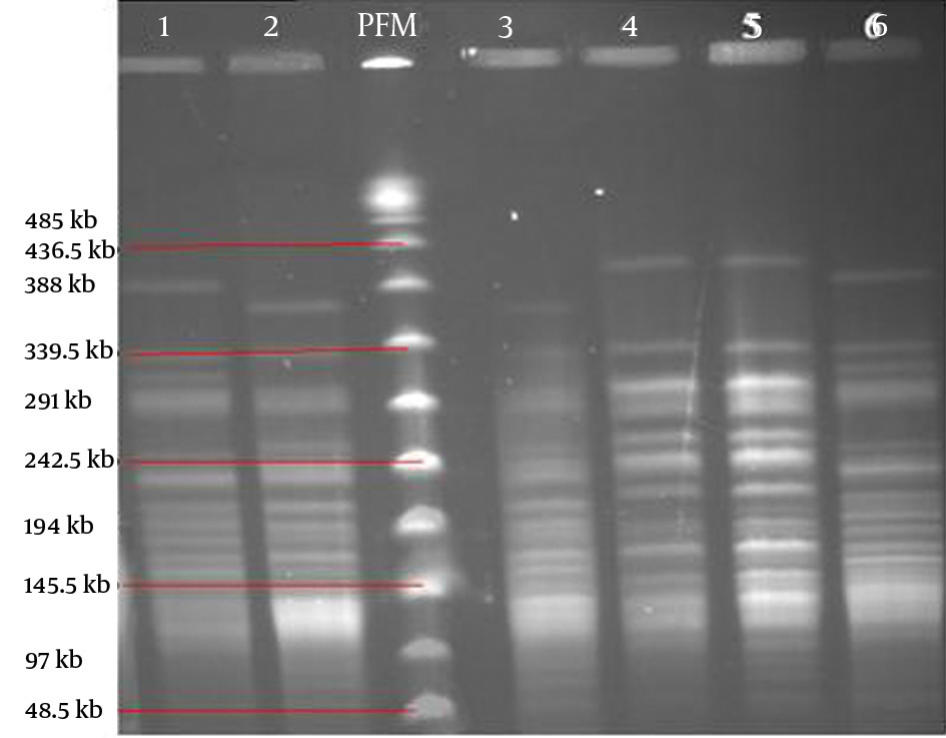
Representative PFGE Profiles of MβLs Producing *P. aeruginosa* The three identical pairs of PFGE patterns: lanes 1 and 6; 2 and 3; 4 and 5. PFM; is Pulsed-Field Marker.

Using PFGE method, we detected isolates with bands between 10 and 16, of which those with 13-14 bands were more prevalent (48.3%). The molecular weights of the observed bands were within the range of 40 kbp and 460 kbp. Thirty seven PFGE profiles were obtained from the digested genome of PA strains, and were numbered from P1 to P37 (Figure 1). P4 and P34 were the most frequent patterns (n = 14, 23.3%). After analyzing the plotted dendrogarm, 43 (72%) and 38 (63%) of the samples showed 80% and 85 % similarity, respectively (Figure 1). Figure 2 shows three identical pairs representative of PFGE profiles.

## 5. Discussion

PA is a gram negative bacterium associated with hospital infections and is often difficult to eradicate due to quick acquisition of antibiotic resistance. Detection of MβLs in PA is crucial for successful treatment of the burn patients and control of the antibiotic resistance. There are several reports on MβLs producing PA, from North and South America, South East of Asia and European countries (20-26). Strains producing these enzymes have been responsible for prolonged nosocomial outbreaks that were accompanied by serious infections (27). Emerging MβLs isolates in hospital settings can pose a therapeutic challenge as well as a serious concern for infection-control managers. In the present study, most patients were young adults, with predominantly accidental burns in males and suicidal burns among rural women, consistent with previous reports (28, 29). High frequency of burns in young adults has been reported worldwide as well as from Iran (30-35). High frequency of burns among young males could be due to carelessness or unprotected working conditions. High incidence of suicides especially among young women in rural areas has been mainly attributed to familial conflicts and social isolation (29, 36).

High similarities (80% to 85%) in the majority of carbapenem resistant pseudomonas have been noticed before. It has been proposed that the isolates with > 80% similarity level can be considered as closely related (37). Comparison of the genetic clustering of the 60 isolates showed a high degree of resemblance by PFGE. These results indicate that a limited number of PA types are associated with infections in our burn centers. A close genetic relationship among strains showed the distribution of organisms in the hospital environment studied. Although some isolates belonged to different clustering groups, they showed more than 80% similarity indicating these isolates originated from a limited number of primary clones. These isolates might tolerate genetic divergence arising from point mutations, insertion or deletion of chromosomal DNA.

The highest percentage of strains with 13 bands formed 26.6 % of the samples, and the least number of strains with 16 bands formed 6.6% of samples. The types P4 and P34 with 7 strains hold within themselves most strains with the same genetic patterns. Our results were comparable to the previous studies (38-41). The similarities of the bands and patterns among different isolates can help in detection of the location of dominant common genes of the pathogenic strains (6). Among the β-lactams currently available, carbapenems are the most effective antibiotics against *P. aeruginosa*. Previously, we showed that burn wounds infected with *P. aeruginosa*, were resistant to most of antibiotics with reduced sensitivity to cabapenems (11). But in another study carried out in Tehran hospitals, the rate of resistance to imipenem was low (40). A high incidence of multidrug resistant (MDR) strains was also detected among our isolates. Most patients in this study had third-degree burns on 18-74% of their total body surface area (BSA), leading to prolonged hospital stay and increased use of antibiotics, which in turn causes increasing emergence of MDR in the hospital. In addition, high quantity administration of antibiotics to treat burn victims could accelerate genetic diversion of a few resistant isolates to overcome antibiotic pressure. Cross-contamination of the burn victims could occur mostly when scrubbing the lesions in the bathroom. To the best of our knowledge, this is the first report documenting the use of PFGE genotyping method to determine the genetic patterns and antibiotic resistance among MβLs producing PA in south of Iran. According to PFGE analysis, a limited number of MRPA can infect burn wounds. Antibiotic susceptibility tests have shown that none of the tested antibiotics is safe to be used for the patients of this center. Cross-contamination and colonization of the wounds with resistant strains could be facilitated by high dose antibiotics in burn centers. Since the present study addressed the genetic analysis of the strains and respective antibiotic resistance in a single burn hospital, extensive and regular surveillance and monitoring of the resistance strains regarding their molecular epidemiology in various burn centers over different time periods is suggested.
